# Supplementary material for: Detailed molecular and epigenetic characterization of the pig IPEC-J2 and chicken SL-29 cell lines
Source: iScience. 2023 Feb 20;26(3):106252. doi: 10.1016/j.isci.2023.106252 (PMC10018572; doi:10.1016/j.isci.2023.106252)
Supplement: Data S1. Complete homer output for identified motifs in Pig IPECJ-2, related to Table 2 — Homer motif analysis results for histone modifications H3K4me1, H3K4me3, H3K27ac, and enhancer elements of pig IPECJ2 cell line. P-values >1e-10 are possible false positives. Within each folder (e.g. peak_files_CTCF) are the html files showing the identified motifs when using homer (e.g. homerResults.html). [file mmc2.zip › S5/Pig_IPECJ_2/peak_files_enhancer/knownResults.html]

peak\_files\_enhancer - Homer Known Motif Enrichment Results


# Homer Known Motif Enrichment Results (peak\_files\_enhancer)

Homer *de novo* Motif Results  
Gene Ontology Enrichment Results  
Known Motif Enrichment Results (txt file)  
Total Target Sequences = 36630, Total Background Sequences = 36571

|  |  |  |  |  |  |  |  |  |  |  |  |
| --- | --- | --- | --- | --- | --- | --- | --- | --- | --- | --- | --- |
| Rank | Motif | Name | P-value | log P-pvalue | q-value (Benjamini) | # Target Sequences with Motif | % of Targets Sequences with Motif | # Background Sequences with Motif | % of Background Sequences with Motif | Motif File | SVG |
| 1 | C T A G T C G A C G A T A C T G C G T A T A C G A G C T T G A C G C T A A C G T G A T C T A G C | Fosl2(bZIP)/3T3L1-Fosl2-ChIP-Seq(GSE56872)/Homer | 1e-439 | -1.012e+03 | 0.0000 | 3326.0 | 9.08% | 1397.0 | 3.82% | motif file (matrix) | svg |
| 2 | C A T G C T A G T C G A A C G T A C T G C G T A T A G C C G A T T G A C C G T A A G C T G A T C | Fra2(bZIP)/Striatum-Fra2-ChIP-Seq(GSE43429)/Homer | 1e-431 | -9.927e+02 | 0.0000 | 4533.0 | 12.37% | 2229.1 | 6.09% | motif file (matrix) | svg |
| 3 | C T A G T C G A A C G T A C T G C G T A T A G C C G A T G T A C C G T A A G C T G A T C G T A C | Jun-AP1(bZIP)/K562-cJun-ChIP-Seq(GSE31477)/Homer | 1e-424 | -9.777e+02 | 0.0000 | 2588.0 | 7.06% | 960.5 | 2.63% | motif file (matrix) | svg |
| 4 | C T A G T C G A G C A T C A T G G C T A T A G C C G A T G T A C C T G A A G C T | JunB(bZIP)/DendriticCells-Junb-ChIP-Seq(GSE36099)/Homer | 1e-422 | -9.733e+02 | 0.0000 | 5059.0 | 13.81% | 2626.2 | 7.18% | motif file (matrix) | svg |
| 5 | A C T G C A T G T C G A A C G T A C T G C G T A A T C G A C G T G T A C C G T A G A C T A G T C | Fos(bZIP)/TSC-Fos-ChIP-Seq(GSE110950)/Homer | 1e-416 | -9.600e+02 | 0.0000 | 5397.0 | 14.73% | 2889.3 | 7.90% | motif file (matrix) | svg |
| 6 | A C T G C T A G T C G A C G A T C A T G G C T A A T C G C G A T G T A C G C T A A G C T G T A C | Fra1(bZIP)/BT549-Fra1-ChIP-Seq(GSE46166)/Homer | 1e-403 | -9.282e+02 | 0.0000 | 5182.0 | 14.14% | 2764.0 | 7.56% | motif file (matrix) | svg |
| 7 | C T A G T C G A A C G T A C T G C G T A A T G C A C G T G T A C C G T A A G C T G A T C G T A C | Atf3(bZIP)/GBM-ATF3-ChIP-Seq(GSE33912)/Homer | 1e-395 | -9.118e+02 | 0.0000 | 5892.0 | 16.08% | 3318.0 | 9.07% | motif file (matrix) | svg |
| 8 | C A G T T G C A A C G T A C T G C G T A A T C G C G A T T G A C C G T A A C G T | BATF(bZIP)/Th17-BATF-ChIP-Seq(GSE39756)/Homer | 1e-386 | -8.901e+02 | 0.0000 | 5757.0 | 15.71% | 3239.0 | 8.86% | motif file (matrix) | svg |
| 9 | T C G A A C G T C A T G G C T A T A G C C G A T G T A C G C T A A C G T A T G C | AP-1(bZIP)/ThioMac-PU.1-ChIP-Seq(GSE21512)/Homer | 1e-385 | -8.872e+02 | 0.0000 | 6285.0 | 17.15% | 3651.7 | 9.98% | motif file (matrix) | svg |
| 10 | C G A T T A C G T G A C G A C T C A T G C G T A T A C G A C G T G T A C C T G A | Bach2(bZIP)/OCILy7-Bach2-ChIP-Seq(GSE44420)/Homer | 1e-208 | -4.792e+02 | 0.0000 | 1819.0 | 4.96% | 811.0 | 2.22% | motif file (matrix) | svg |
| 11 | G A T C G T A C C G A T A C T G A C T G C G T A C G T A A C G T A C T G G A T C | TEAD(TEA)/Fibroblast-PU.1-ChIP-Seq(Unpublished)/Homer | 1e-143 | -3.297e+02 | 0.0000 | 5230.0 | 14.27% | 3673.3 | 10.04% | motif file (matrix) | svg |
| 12 | T A G C T A G C G C A T C A T G A C T G G C T A C G T A A C G T A C T G G A T C | TEAD4(TEA)/Tropoblast-Tead4-ChIP-Seq(GSE37350)/Homer | 1e-124 | -2.865e+02 | 0.0000 | 5718.0 | 15.61% | 4192.4 | 11.46% | motif file (matrix) | svg |
| 13 | T G A C A G T C C T G A T G A C C G T A A C G T A C G T A G T C A G T C C G T A | TEAD1(TEAD)/HepG2-TEAD1-ChIP-Seq(Encode)/Homer | 1e-122 | -2.815e+02 | 0.0000 | 7078.0 | 19.32% | 5406.2 | 14.78% | motif file (matrix) | svg |
| 14 | A T G C G A T C C G A T C T A G A C T G G C T A C G T A A G C T A C T G A G C T | TEAD2(TEA)/Py2T-Tead2-ChIP-Seq(GSE55709)/Homer | 1e-104 | -2.409e+02 | 0.0000 | 3707.0 | 10.12% | 2572.0 | 7.03% | motif file (matrix) | svg |
| 15 | T A C G T C G A C A G T A C T G G C T A A T G C C G A T G T A C C G T A A C T G T A G C C G T A | NF-E2(bZIP)/K562-NFE2-ChIP-Seq(GSE31477)/Homer | 1e-98 | -2.263e+02 | 0.0000 | 655.0 | 1.79% | 253.2 | 0.69% | motif file (matrix) | svg |
| 16 | A G C T C T A G T G A C C G T A A C G T C G A T A G T C A G T C C T G A C A T G | TEAD3(TEA)/HepG2-TEAD3-ChIP-Seq(Encode)/Homer | 1e-95 | -2.193e+02 | 0.0000 | 7923.0 | 21.63% | 6362.0 | 17.39% | motif file (matrix) | svg |
| 17 | G T C A G C A T A C T G G T A C G A C T A C T G G C T A A T C G C A G T G T A C C G T A A G C T | Nrf2(bZIP)/Lymphoblast-Nrf2-ChIP-Seq(GSE37589)/Homer | 1e-82 | -1.905e+02 | 0.0000 | 581.0 | 1.59% | 231.8 | 0.63% | motif file (matrix) | svg |
| 18 | T C G A T A G C G T C A A C T G A C T G C G T A C G T A C T A G A G C T T C A G | ERG(ETS)/VCaP-ERG-ChIP-Seq(GSE14097)/Homer | 1e-74 | -1.714e+02 | 0.0000 | 9164.0 | 25.01% | 7691.0 | 21.03% | motif file (matrix) | svg |
| 19 | C G T A C G T A C G T A G C A T G C A T T A C G G T A C G A C T A C T G C G T A A T C G A C G T G T A C C G T A A G C T | Bach1(bZIP)/K562-Bach1-ChIP-Seq(GSE31477)/Homer | 1e-70 | -1.613e+02 | 0.0000 | 634.0 | 1.73% | 286.5 | 0.78% | motif file (matrix) | svg |
| 20 | A T G C C T G A A T C G T A C G A G T C C G A T T C A G C G A T C T A G A G C T G T C A G T C A C G T A A G T C C G T A T A C G C T G A | Fox:Ebox(Forkhead,bHLH)/Panc1-Foxa2-ChIP-Seq(GSE47459)/Homer | 1e-62 | -1.447e+02 | 0.0000 | 5787.0 | 15.80% | 4672.8 | 12.78% | motif file (matrix) | svg |
| 21 | C T G A T A G C T G A C T C A G C T A G G T C A C G T A T C A G A G C T T C A G | ETV4(ETS)/HepG2-ETV4-ChIP-Seq(ENCODE)/Homer | 1e-61 | -1.423e+02 | 0.0000 | 5549.0 | 15.15% | 4464.9 | 12.21% | motif file (matrix) | svg |
| 22 | A T G C A G T C C T G A A G T C C G A T A C G T A G T C A G T C A C G T A T C G G A C T A C G T | Etv2(ETS)/ES-ER71-ChIP-Seq(GSE59402)/Homer | 1e-60 | -1.392e+02 | 0.0000 | 5409.0 | 14.76% | 4348.4 | 11.89% | motif file (matrix) | svg |
| 23 | C G T A C G T A C G T A G C A T G C A T A C T G G T A C G A C T C T A G G C T A T A C G G A C T T G A C C G T A A G C T | NFE2L2(bZIP)/HepG2-NFE2L2-ChIP-Seq(Encode)/Homer | 1e-59 | -1.368e+02 | 0.0000 | 651.0 | 1.78% | 319.7 | 0.87% | motif file (matrix) | svg |
| 24 | A G T C C T G A A G T C C G A T C A G T G A T C A T G C A C T G A T C G G A C T | Fli1(ETS)/CD8-FLI-ChIP-Seq(GSE20898)/Homer | 1e-54 | -1.262e+02 | 0.0000 | 5925.0 | 16.17% | 4869.8 | 13.31% | motif file (matrix) | svg |
| 25 | T C G A T A G C T G C A A C T G A C T G C G T A C G T A C T A G G A C T T A C G | ETS1(ETS)/Jurkat-ETS1-ChIP-Seq(GSE17954)/Homer | 1e-53 | -1.236e+02 | 0.0000 | 5749.0 | 15.69% | 4718.7 | 12.90% | motif file (matrix) | svg |
| 26 | T C G A A G C T A C G T A C G T A G T C A G T C A C G T A T C G G A C T A T C G | EWS:ERG-fusion(ETS)/CADO\_ES1-EWS:ERG-ChIP-Seq(SRA014231)/Homer | 1e-48 | -1.121e+02 | 0.0000 | 4964.0 | 13.55% | 4045.1 | 11.06% | motif file (matrix) | svg |
| 27 | T C A G T A G C G A C T C A T G C T G A A T C G G C A T G T A C C G T A A C T G T A G C T G C A | MafK(bZIP)/C2C12-MafK-ChIP-Seq(GSE36030)/Homer | 1e-48 | -1.112e+02 | 0.0000 | 1387.0 | 3.79% | 914.2 | 2.50% | motif file (matrix) | svg |
| 28 | A T G C G A C T A C G T C T A G A C G T A C G T A C G T C T G A G A T C G C T A A G C T C G T A | Foxa2(Forkhead)/Liver-Foxa2-ChIP-Seq(GSE25694)/Homer | 1e-47 | -1.092e+02 | 0.0000 | 5232.0 | 14.28% | 4302.6 | 11.76% | motif file (matrix) | svg |
| 29 | T G C A C T G A A G T C G T C A A C T G A C T G C G T A C G T A C T G A A G C T | EWS:FLI1-fusion(ETS)/SK\_N\_MC-EWS:FLI1-ChIP-Seq(SRA014231)/Homer | 1e-44 | -1.023e+02 | 0.0000 | 3086.0 | 8.42% | 2389.5 | 6.53% | motif file (matrix) | svg |
| 30 | T G C A C T G A A T G C G T C A A C T G A C T G C G T A C G T A C T A G A G C T | Ets1-distal(ETS)/CD4+-PolII-ChIP-Seq(Barski\_et\_al.)/Homer | 1e-43 | -1.004e+02 | 0.0000 | 1839.0 | 5.02% | 1312.1 | 3.59% | motif file (matrix) | svg |
| 31 | T C G A C T G A T A G C T G A C T C A G T C A G C G T A C G T A T C A G A G C T | ETV1(ETS)/GIST48-ETV1-ChIP-Seq(GSE22441)/Homer | 1e-36 | -8.495e+01 | 0.0000 | 7751.0 | 21.16% | 6773.5 | 18.52% | motif file (matrix) | svg |
| 32 | G C T A T C G A C G T A C T A G A G C T G T C A G T C A C G T A A G T C C G T A | FOXA1(Forkhead)/LNCAP-FOXA1-ChIP-Seq(GSE27824)/Homer | 1e-36 | -8.379e+01 | 0.0000 | 8652.0 | 23.61% | 7637.6 | 20.88% | motif file (matrix) | svg |
| 33 | C T A G G T A C A G T C T G C A A G T C C T G A A G T C A G T C A G T C G C T A | Klf4(Zf)/mES-Klf4-ChIP-Seq(GSE11431)/Homer | 1e-33 | -7.743e+01 | 0.0000 | 2180.0 | 5.95% | 1669.0 | 4.56% | motif file (matrix) | svg |
| 34 | A G T C T A G C G A C T A C G T C T A G A C G T A C G T A C G T C T G A A G T C G C T A G A C T C G T A C T A G A C T G | Foxa3(Forkhead)/Liver-Foxa3-ChIP-Seq(GSE77670)/Homer | 1e-32 | -7.525e+01 | 0.0000 | 2271.0 | 6.20% | 1756.4 | 4.80% | motif file (matrix) | svg |
| 35 | C T G A A G T C C G A T A G C T A T G C G T A C A C G T A T C G C A G T G C A T | Elf4(ETS)/BMDM-Elf4-ChIP-Seq(GSE88699)/Homer | 1e-32 | -7.459e+01 | 0.0000 | 5834.0 | 15.92% | 5022.2 | 13.73% | motif file (matrix) | svg |
| 36 | G C T A A T C G G C T A G A C T G C T A T C G A T A G C T C G A | GATA3(Zf)/iTreg-Gata3-ChIP-Seq(GSE20898)/Homer | 1e-31 | -7.290e+01 | 0.0000 | 9547.0 | 26.06% | 8564.1 | 23.42% | motif file (matrix) | svg |
| 37 | A C G T C T A G A G C T A C G T A C G T C T G A A G T C G A C T A G C T C G T A | FOXM1(Forkhead)/MCF7-FOXM1-ChIP-Seq(GSE72977)/Homer | 1e-31 | -7.225e+01 | 0.0000 | 7529.0 | 20.55% | 6637.0 | 18.15% | motif file (matrix) | svg |
| 38 | C G T A C T A G A C T G A C T G G A C T C T A G C A G T C T A G C A T G G A T C | KLF5(Zf)/LoVo-KLF5-ChIP-Seq(GSE49402)/Homer | 1e-31 | -7.205e+01 | 0.0000 | 6480.0 | 17.69% | 5644.3 | 15.43% | motif file (matrix) | svg |
| 39 | T C G A T C G A T A G C G T A C T C A G T A C G C G T A C G T A T C A G A G C T | GABPA(ETS)/Jurkat-GABPa-ChIP-Seq(GSE17954)/Homer | 1e-31 | -7.189e+01 | 0.0000 | 4710.0 | 12.86% | 3987.1 | 10.90% | motif file (matrix) | svg |
| 40 | C G T A T G A C T A G C T G C A A C T G A C T G C G T A C G T A T C A G G A C T | ELF3(ETS)/PDAC-ELF3-ChIP-Seq(GSE64557)/Homer | 1e-30 | -6.970e+01 | 0.0000 | 4596.0 | 12.54% | 3892.7 | 10.64% | motif file (matrix) | svg |
| 41 | G C T A T C G A C G T A C T A G A G C T G T C A G T C A C G T A A G T C C G T A | FOXA1(Forkhead)/MCF7-FOXA1-ChIP-Seq(GSE26831)/Homer | 1e-30 | -6.913e+01 | 0.0000 | 7453.0 | 20.34% | 6584.1 | 18.00% | motif file (matrix) | svg |
| 42 | C G A T C T A G A C G T G T C A C G T A C G T A A G T C C G T A | Foxo3(Forkhead)/U2OS-Foxo3-ChIP-Seq(E-MTAB-2701)/Homer | 1e-29 | -6.903e+01 | 0.0000 | 4814.0 | 13.14% | 4098.9 | 11.21% | motif file (matrix) | svg |
| 43 | C G T A T A C G T C G A A C T G A C T G C G T A C G T A T A C G A G C T T A C G | PU.1(ETS)/ThioMac-PU.1-ChIP-Seq(GSE21512)/Homer | 1e-28 | -6.479e+01 | 0.0000 | 2858.0 | 7.80% | 2319.3 | 6.34% | motif file (matrix) | svg |
| 44 | G A T C T C G A A G T C C G A T C G A T A G T C A T G C A C T G A T C G G A C T | Elk1(ETS)/Hela-Elk1-ChIP-Seq(GSE31477)/Homer | 1e-27 | -6.275e+01 | 0.0000 | 2520.0 | 6.88% | 2022.3 | 5.53% | motif file (matrix) | svg |
| 45 | C G T A T A G C T A G C T G C A A C T G C T A G C G T A C G T A T C A G G A C T | EHF(ETS)/LoVo-EHF-ChIP-Seq(GSE49402)/Homer | 1e-26 | -6.171e+01 | 0.0000 | 7674.0 | 20.95% | 6843.6 | 18.71% | motif file (matrix) | svg |
| 46 | A G T C A C G T A C T G A G C T A C G T A C G T G T C A A G T C | Foxo1(Forkhead)/RAW-Foxo1-ChIP-Seq(Fan\_et\_al.)/Homer | 1e-26 | -6.169e+01 | 0.0000 | 10596.0 | 28.92% | 9658.5 | 26.41% | motif file (matrix) | svg |
| 47 | G A C T T C A G C T A G A G T C A G T C G T A C A G T C C T G A A G T C A G T C A G T C G A C T A G T C A C T G A T G C | KLF3(Zf)/MEF-Klf3-ChIP-Seq(GSE44748)/Homer | 1e-26 | -6.048e+01 | 0.0000 | 2272.0 | 6.20% | 1808.4 | 4.94% | motif file (matrix) | svg |
| 48 | T G C A T C G A T A G C G T A C T C A G C T A G G T C A G C T A T C A G G A C T | ETS(ETS)/Promoter/Homer | 1e-26 | -6.015e+01 | 0.0000 | 1452.0 | 3.96% | 1086.4 | 2.97% | motif file (matrix) | svg |
| 49 | A G C T T G A C C G A T C G A T C T A G A C G T C A G T C A G T G C T A A G T C | FOXK1(Forkhead)/HEK293-FOXK1-ChIP-Seq(GSE51673)/Homer | 1e-26 | -6.011e+01 | 0.0000 | 6659.0 | 18.18% | 5886.8 | 16.10% | motif file (matrix) | svg |
| 50 | C G T A C T G A C T A G C T G A A G T C G C T A C G A T A T C G G A C T G A T C A G T C C T G A C T A G C T A G A G T C G C T A C G A T C T A G G A T C G A T C | p73(p53)/Trachea-p73-ChIP-Seq(PRJNA310161)/Homer | 1e-25 | -5.888e+01 | 0.0000 | 281.0 | 0.77% | 139.4 | 0.38% | motif file (matrix) | svg |
| 51 | A G C T A G T C A G T C A C G T C T A G A C G T A C G T A C G T C G T A A G T C G A T C C G T A | FOXP1(Forkhead)/H9-FOXP1-ChIP-Seq(GSE31006)/Homer | 1e-25 | -5.884e+01 | 0.0000 | 2821.0 | 7.70% | 2310.8 | 6.32% | motif file (matrix) | svg |
| 52 | G A T C C T G A A G T C C G A T C G A T G A T C A G T C A C T G A T C G A G C T | Elk4(ETS)/Hela-Elk4-ChIP-Seq(GSE31477)/Homer | 1e-24 | -5.656e+01 | 0.0000 | 2546.0 | 6.95% | 2070.1 | 5.66% | motif file (matrix) | svg |
| 53 | C T G A T G C A T A G C T G A C T A C G T C A G C T G A G C T A T C A G G A C T | ELF1(ETS)/Jurkat-ELF1-ChIP-Seq(SRA014231)/Homer | 1e-23 | -5.315e+01 | 0.0000 | 2233.0 | 6.09% | 1801.2 | 4.92% | motif file (matrix) | svg |
| 54 | C G T A A C T G C G T A A C G T C G T A C T G A T C A G T G C A A G C T T G A C | TRPS1(Zf)/MCF7-TRPS1-ChIP-Seq(GSE107013)/Homer | 1e-22 | -5.285e+01 | 0.0000 | 12708.0 | 34.69% | 11790.6 | 32.24% | motif file (matrix) | svg |
| 55 | A G T C C T A G C T A G A G T C G A T C G T A C A G T C C T A G A G T C A G T C A G T C G T A C | Sp2(Zf)/HEK293-Sp2.eGFP-ChIP-Seq(Encode)/Homer | 1e-22 | -5.246e+01 | 0.0000 | 6834.0 | 18.65% | 6104.2 | 16.69% | motif file (matrix) | svg |
| 56 | G A C T C G A T T C A G G A T C G A C T A G C T A G C T A G T C G A T C C G T A C T A G C T A G T C G A T C G A C T G A | Bcl6(Zf)/Liver-Bcl6-ChIP-Seq(GSE31578)/Homer | 1e-22 | -5.181e+01 | 0.0000 | 8027.0 | 21.91% | 7252.5 | 19.83% | motif file (matrix) | svg |
| 57 | T C A G C T A G C T A G A C T G A C T G G A T C A C T G A C T G C T A G C T A G A G T C G A T C | KLF1(Zf)/HUDEP2-KLF1-CutnRun(GSE136251)/Homer | 1e-22 | -5.126e+01 | 0.0000 | 4129.0 | 11.27% | 3554.5 | 9.72% | motif file (matrix) | svg |
| 58 | A G T C G A C T C A G T G T A C A G T C A T C G T C A G A C T G G T C A C G T A | Stat3(Stat)/mES-Stat3-ChIP-Seq(GSE11431)/Homer | 1e-21 | -4.934e+01 | 0.0000 | 2635.0 | 7.19% | 2182.4 | 5.97% | motif file (matrix) | svg |
| 59 | C G T A G C T A C G A T C T A G A C G T G T C A C G T A C G T A A G T C C G T A T G C A T A C G | FoxL2(Forkhead)/Ovary-FoxL2-ChIP-Seq(GSE60858)/Homer | 1e-21 | -4.874e+01 | 0.0000 | 6038.0 | 16.48% | 5371.3 | 14.69% | motif file (matrix) | svg |
| 60 | A G C T A G T C A T G C A G C T A C G T C G T A A C G T A G T C C G A T A T G C | Gata2(Zf)/K562-GATA2-ChIP-Seq(GSE18829)/Homer | 1e-20 | -4.792e+01 | 0.0000 | 4177.0 | 11.40% | 3618.7 | 9.89% | motif file (matrix) | svg |
| 61 | C T G A A C T G A C T G A G T C A G T C A G C T C T A G T A C G | ZFX(Zf)/mES-Zfx-ChIP-Seq(GSE11431)/Homer | 1e-20 | -4.778e+01 | 0.0000 | 6011.0 | 16.41% | 5352.9 | 14.64% | motif file (matrix) | svg |
| 62 | C T A G T C G A C T G A C G T A T A C G G A C T T C A G T C G A G T C A T G C A T A C G A G C T | IRF2(IRF)/Erythroblas-IRF2-ChIP-Seq(GSE36985)/Homer | 1e-20 | -4.734e+01 | 0.0000 | 736.0 | 2.01% | 510.0 | 1.39% | motif file (matrix) | svg |
| 63 | T A C G A T G C G C T A A C T G C G T A A C G T C G T A C T G A T A C G T C G A | Gata4(Zf)/Heart-Gata4-ChIP-Seq(GSE35151)/Homer | 1e-20 | -4.618e+01 | 0.0000 | 6452.0 | 17.61% | 5784.2 | 15.81% | motif file (matrix) | svg |
| 64 | G T A C C A G T A C T G A C T G A C T G G A T C A C T G A C G T A C T G A C T G A G T C G A T C | KLF6(Zf)/PDAC-KLF6-ChIP-Seq(GSE64557)/Homer | 1e-19 | -4.562e+01 | 0.0000 | 5227.0 | 14.27% | 4622.3 | 12.64% | motif file (matrix) | svg |
| 65 | C T G A A T C G A G C T A G C T A C G T T A G C C T G A T A C G C G A T A C G T G A C T A G T C | ISRE(IRF)/ThioMac-LPS-Expression(GSE23622)/Homer | 1e-19 | -4.463e+01 | 0.0000 | 456.0 | 1.24% | 288.2 | 0.79% | motif file (matrix) | svg |
| 66 | G C T A A G T C T A C G T G C A A T C G T C A G G C T A T C G A T C A G A G C T | ELF5(ETS)/T47D-ELF5-ChIP-Seq(GSE30407)/Homer | 1e-18 | -4.367e+01 | 0.0000 | 4370.0 | 11.93% | 3825.8 | 10.46% | motif file (matrix) | svg |
| 67 | T A G C G C T A A C T G C G T A A C G T C G T A C G T A T A C G T C A G T C G A | Gata1(Zf)/K562-GATA1-ChIP-Seq(GSE18829)/Homer | 1e-18 | -4.315e+01 | 0.0000 | 3660.0 | 9.99% | 3162.9 | 8.65% | motif file (matrix) | svg |
| 68 | C T A G C T G A A G T C G C T A C G A T A C T G G A C T G A T C G A T C C T G A C T A G C T G A T G A C G C T A C G A T T C A G G A C T G A T C G A T C T G A C | p53(p53)/Saos-p53-ChIP-Seq(GSE15780)/Homer | 1e-18 | -4.295e+01 | 0.0000 | 470.0 | 1.28% | 302.0 | 0.83% | motif file (matrix) | svg |
| 69 | C T A G C T G A A G T C G C T A C G A T A C T G G A C T G A T C G A T C C T G A C T A G C T G A T G A C G C T A C G A T T C A G G A C T G A T C G A T C T G A C | p53(p53)/Saos-p53-ChIP-Seq/Homer | 1e-18 | -4.295e+01 | 0.0000 | 470.0 | 1.28% | 302.0 | 0.83% | motif file (matrix) | svg |
| 70 | T A C G T A G C G C T A C G A T C T A G A C G T C A G T C A G T G C T A A G T C G T C A G C A T | FOXK2(Forkhead)/U2OS-FOXK2-ChIP-Seq(E-MTAB-2204)/Homer | 1e-18 | -4.217e+01 | 0.0000 | 3908.0 | 10.67% | 3400.4 | 9.30% | motif file (matrix) | svg |
| 71 | A G C T A T G C G A C T G C A T C G T A A G C T G T A C C G A T A T C G A G T C | Gata6(Zf)/HUG1N-GATA6-ChIP-Seq(GSE51936)/Homer | 1e-18 | -4.149e+01 | 0.0000 | 5954.0 | 16.25% | 5343.5 | 14.61% | motif file (matrix) | svg |
| 72 | T G A C C G A T A C T G A C T G A C T G G A C T A C T G A C G T A C T G A C T G G A T C G A T C | EKLF(Zf)/Erythrocyte-Klf1-ChIP-Seq(GSE20478)/Homer | 1e-16 | -3.905e+01 | 0.0000 | 1385.0 | 3.78% | 1095.0 | 2.99% | motif file (matrix) | svg |
| 73 | A T C G A G T C A G T C G A C T A T G C C T G A C T A G A C T G T A C G G T A C C T G A C G A T | AP-2gamma(AP2)/MCF7-TFAP2C-ChIP-Seq(GSE21234)/Homer | 1e-16 | -3.832e+01 | 0.0000 | 5149.0 | 14.05% | 4600.0 | 12.58% | motif file (matrix) | svg |
| 74 | C T G A C T G A C T G A A T G C G A T C C A T G A C T G G A C T G A C T G C A T C G T A C G T A A G T C G T A C C T G A A T C G G C A T G A C T G A C T A G C T | GRHL2(CP2)/HBE-GRHL2-ChIP-Seq(GSE46194)/Homer | 1e-16 | -3.825e+01 | 0.0000 | 2054.0 | 5.61% | 1702.1 | 4.65% | motif file (matrix) | svg |
| 75 | G C A T G C A T C T G A A C G T C T G A A C G T C G T A C G T A C G T A A G T C G T C A G T C A | Foxf1(Forkhead)/Lung-Foxf1-ChIP-Seq(GSE77951)/Homer | 1e-15 | -3.628e+01 | 0.0000 | 6590.0 | 17.99% | 5994.3 | 16.39% | motif file (matrix) | svg |
| 76 | C G T A G A C T C G A T A T C G G T A C G C A T C A T G C G T A T A C G G C A T G T A C C G T A C A T G A T G C G C T A C T A G G C A T G C A T G C A T G A C T | MafB(bZIP)/BMM-Mafb-ChIP-Seq(GSE75722)/Homer | 1e-15 | -3.570e+01 | 0.0000 | 2236.0 | 6.10% | 1881.6 | 5.14% | motif file (matrix) | svg |
| 77 | G T C A T C G A C T A G C T A G A G T C G T C A C G A T C T A G G A C T G A T C G A T C T C A G C T A G C T G A A G T C G C T A C A G T T C A G G A T C G A T C | p63(p53)/Keratinocyte-p63-ChIP-Seq(GSE17611)/Homer | 1e-13 | -3.168e+01 | 0.0000 | 1432.0 | 3.91% | 1166.1 | 3.19% | motif file (matrix) | svg |
| 78 | A G T C G A C T C A G T A C T G C T A G T G A C G C T A A T G C G C A T A T C G C G A T A C T G G A T C G T A C G T C A C T G A | NF1(CTF)/LNCAP-NF1-ChIP-Seq(Unpublished)/Homer | 1e-12 | -2.957e+01 | 0.0000 | 1898.0 | 5.18% | 1601.5 | 4.38% | motif file (matrix) | svg |
| 79 | C T G A T A G C G C T A C G A T A T C G A G T C G A T C G A T C C T A G T C A G T C A G G T A C G C T A C A G T | p53(p53)/mES-cMyc-ChIP-Seq(GSE11431)/Homer | 1e-11 | -2.740e+01 | 0.0000 | 103.0 | 0.28% | 47.2 | 0.13% | motif file (matrix) | svg |
| 80 | T C A G C T G A C G T A C G T A T A C G G C A T C T A G C T G A C G T A C G T A T A C G G A C T | IRF1(IRF)/PBMC-IRF1-ChIP-Seq(GSE43036)/Homer | 1e-11 | -2.720e+01 | 0.0000 | 1065.0 | 2.91% | 854.3 | 2.34% | motif file (matrix) | svg |
| 81 | G C T A C G A T C A T G A T G C A G T C A G T C G A C T T A C G T C G A C T A G A C T G T A G C | AP-2alpha(AP2)/Hela-AP2alpha-ChIP-Seq(GSE31477)/Homer | 1e-11 | -2.658e+01 | 0.0000 | 3952.0 | 10.79% | 3549.9 | 9.71% | motif file (matrix) | svg |
| 82 | C A G T A C T G C G T A G T C A C G T A A G C T G A T C A G C T A C T G C G T A A G T C G T C A A G T C A G T C A T C G | Six4(Homeobox)/MCF7-SIX4-ChIP-Seq(Encode)/Homer | 1e-11 | -2.584e+01 | 0.0000 | 295.0 | 0.81% | 193.2 | 0.53% | motif file (matrix) | svg |
| 83 | G A C T C T A G G A T C C A G T A C T G C T G A A T G C G C A T A T G C C T G A | MafA(bZIP)/Islet-MafA-ChIP-Seq(GSE30298)/Homer | 1e-11 | -2.584e+01 | 0.0000 | 4321.0 | 11.79% | 3907.3 | 10.68% | motif file (matrix) | svg |
| 84 | A T G C T C G A A G T C A G C T A C G T G T A C A G T C G C T A C T A G C A T G G T C A C T G A T C A G A G T C | Stat3+il21(Stat)/CD4-Stat3-ChIP-Seq(GSE19198)/Homer | 1e-11 | -2.561e+01 | 0.0000 | 3656.0 | 9.98% | 3275.9 | 8.96% | motif file (matrix) | svg |
| 85 | A T G C T C A G T C G A G C A T A C T G C G T A A G T C T C A G G A C T T G A C C G T A A G C T | Atf2(bZIP)/3T3L1-Atf2-ChIP-Seq(GSE56872)/Homer | 1e-10 | -2.529e+01 | 0.0000 | 1585.0 | 4.33% | 1335.3 | 3.65% | motif file (matrix) | svg |
| 86 | G T A C G C T A T C A G C T G A C T A G C A T G A G C T G A T C T G C A T C G A C T G A A C T G C A G T A G T C G A T C G C T A | HNF4a(NR),DR1/HepG2-HNF4a-ChIP-Seq(GSE25021)/Homer | 1e-10 | -2.366e+01 | 0.0000 | 2569.0 | 7.01% | 2262.0 | 6.18% | motif file (matrix) | svg |
| 87 | T C A G A G C T A C G T A C G T G T A C G A T C C G T A C T A G C A T G G T C A C G T A T C G A | STAT4(Stat)/CD4-Stat4-ChIP-Seq(GSE22104)/Homer | 1e-10 | -2.341e+01 | 0.0000 | 5659.0 | 15.45% | 5215.8 | 14.26% | motif file (matrix) | svg |
| 88 | T C A G C T A G C T A G T G C A C G A T C G A T C G T A C T A G | GSC(Homeobox)/FrogEmbryos-GSC-ChIP-Seq(DRA000576)/Homer | 1e-10 | -2.322e+01 | 0.0000 | 7384.0 | 20.15% | 6890.0 | 18.84% | motif file (matrix) | svg |
| 89 | C T A G C A T G T G C A A C G T A G T C C G T A C A T G T C A G A C G T A C G T G C T A A G T C | Six1(Homeobox)/Myoblast-Six1-ChIP-Chip(GSE20150)/Homer | 1e-9 | -2.126e+01 | 0.0000 | 1370.0 | 3.74% | 1158.8 | 3.17% | motif file (matrix) | svg |
| 90 | T C G A T C G A T A C G G A T C G T C A G T A C C G A T A G C T G T C A T G C A | Nkx3.1(Homeobox)/LNCaP-Nkx3.1-ChIP-Seq(GSE28264)/Homer | 1e-9 | -2.104e+01 | 0.0000 | 11756.0 | 32.09% | 11200.9 | 30.62% | motif file (matrix) | svg |
| 91 | T G C A A C T G T A C G A T G C A G T C G A C T T C G A A T C G | ZNF711(Zf)/SHSY5Y-ZNF711-ChIP-Seq(GSE20673)/Homer | 1e-9 | -2.088e+01 | 0.0000 | 8613.0 | 23.51% | 8116.0 | 22.19% | motif file (matrix) | svg |
| 92 | T A G C G T A C C G T A C T A G A C T G T G C A C G T A A T G C C G T A A T C G | AR-halfsite(NR)/LNCaP-AR-ChIP-Seq(GSE27824)/Homer | 1e-9 | -2.080e+01 | 0.0000 | 18100.0 | 49.40% | 17494.1 | 47.83% | motif file (matrix) | svg |
| 93 | T G C A A G C T C T G A A T C G G A C T C T A G G T A C G A T C G T C A A G T C G T A C G A C T C T A G A T C G G C A T C A T G C A T G G A T C G T A C C T G A | CTCF(Zf)/CD4+-CTCF-ChIP-Seq(Barski\_et\_al.)/Homer | 1e-8 | -2.055e+01 | 0.0000 | 491.0 | 1.34% | 370.9 | 1.01% | motif file (matrix) | svg |
| 94 | T C A G T C A G G C T A C G T A T A C G G A C T T C A G T C G A C T G A C G T A T A C G G A C T | IRF8(IRF)/BMDM-IRF8-ChIP-Seq(GSE77884)/Homer | 1e-8 | -2.050e+01 | 0.0000 | 2078.0 | 5.67% | 1821.5 | 4.98% | motif file (matrix) | svg |
| 95 | A T C G G A T C G C A T C G T A G T C A A C G T A T G C A G T C | CRX(Homeobox)/Retina-Crx-ChIP-Seq(GSE20012)/Homer | 1e-8 | -2.048e+01 | 0.0000 | 13696.0 | 37.38% | 13124.6 | 35.88% | motif file (matrix) | svg |
| 96 | T C G A G C A T A C T G C T G A A G T C T C A G G A C T G T A C C G T A A G C T A G T C G A T C | c-Jun-CRE(bZIP)/K562-cJun-ChIP-Seq(GSE31477)/Homer | 1e-8 | -1.977e+01 | 0.0000 | 1517.0 | 4.14% | 1302.7 | 3.56% | motif file (matrix) | svg |
| 97 | C G A T C T A G A C G T A C G T A C G T C G T A A G C T C G A T A G C T C G T A C T A G T A G C | FoxD3(forkhead)/ZebrafishEmbryo-Foxd3.biotin-ChIP-seq(GSE106676)/Homer | 1e-8 | -1.931e+01 | 0.0000 | 6140.0 | 16.76% | 5725.4 | 15.65% | motif file (matrix) | svg |
| 98 | T G A C C G A T C T G A C T A G C T A G A C G T A T G C T G C A T C G A C T G A C T A G C A T G A C G T A G T C C G T A | PPARa(NR),DR1/Liver-Ppara-ChIP-Seq(GSE47954)/Homer | 1e-8 | -1.908e+01 | 0.0000 | 6888.0 | 18.80% | 6455.0 | 17.65% | motif file (matrix) | svg |
| 99 | C T A G C T A G C G T A C G T A T A C G C G A T C T A G C T G A C T G A C G T A T A C G G A C T | PU.1:IRF8(ETS:IRF)/pDC-Irf8-ChIP-Seq(GSE66899)/Homer | 1e-7 | -1.840e+01 | 0.0000 | 1194.0 | 3.26% | 1011.3 | 2.76% | motif file (matrix) | svg |
| 100 | C T G A T C A G C A G T C T A G A C T G C T A G G A T C A T C G A C T G C T G A T C A G G A T C | Sp5(Zf)/mES-Sp5.Flag-ChIP-Seq(GSE72989)/Homer | 1e-7 | -1.839e+01 | 0.0000 | 4096.0 | 11.18% | 3759.3 | 10.28% | motif file (matrix) | svg |
| 101 | T C G A A C G T A C T G C T G A A G T C T C A G A G C T G T A C C G T A A G C T G A T C T C G A | JunD(bZIP)/K562-JunD-ChIP-Seq/Homer | 1e-7 | -1.822e+01 | 0.0000 | 387.0 | 1.06% | 287.6 | 0.79% | motif file (matrix) | svg |
| 102 | A G T C C G A T A C T G A T C G T G A C G C T A C A T G A T C G T G A C C G A T A C T G T A G C G T A C G T C A | Tlx?(NR)/NPC-H3K4me1-ChIP-Seq(GSE16256)/Homer | 1e-7 | -1.801e+01 | 0.0000 | 1945.0 | 5.31% | 1714.0 | 4.69% | motif file (matrix) | svg |
| 103 | A G C T G A C T C T A G C G T A C A T G C G A T C T A G A T C G G A C T C A G T | Bapx1(Homeobox)/VertebralCol-Bapx1-ChIP-Seq(GSE36672)/Homer | 1e-7 | -1.719e+01 | 0.0000 | 10204.0 | 27.85% | 9728.0 | 26.60% | motif file (matrix) | svg |
| 104 | C T A G C G T A G T C A C G T A A G T C G A T C A G C T C T A G C G T A A C G T G T C A G A T C | Six2(Homeobox)/NephronProgenitor-Six2-ChIP-Seq(GSE39837)/Homer | 1e-7 | -1.704e+01 | 0.0000 | 5787.0 | 15.80% | 5409.1 | 14.79% | motif file (matrix) | svg |
| 105 | C T A G C A T G C A T G T A C G A G T C G C A T A G C T C T A G A C G T A G T C G A C T A C T G A C T G A C T G T C G A | Zfp809(Zf)/ES-Zfp809-ChIP-Seq(GSE70799)/Homer | 1e-7 | -1.687e+01 | 0.0000 | 1032.0 | 2.82% | 870.6 | 2.38% | motif file (matrix) | svg |
| 106 | C T A G T A C G A G T C C G T A A G T C A C G T A G T C T C G A C G T A T A C G | Nkx2.1(Homeobox)/LungAC-Nkx2.1-ChIP-Seq(GSE43252)/Homer | 1e-7 | -1.630e+01 | 0.0000 | 13616.0 | 37.16% | 13111.5 | 35.85% | motif file (matrix) | svg |
| 107 | C G A T C T G A C G T A C A G T A G T C G A T C G A T C A C T G | Pitx1(Homeobox)/Chicken-Pitx1-ChIP-Seq(GSE38910)/Homer | 1e-6 | -1.571e+01 | 0.0000 | 23579.0 | 64.36% | 23067.0 | 63.07% | motif file (matrix) | svg |
| 108 | T C A G A G C T A T G C C G T A A G C T T C A G C A G T A C T G C T G A A G T C | MITF(bHLH)/MastCells-MITF-ChIP-Seq(GSE48085)/Homer | 1e-6 | -1.559e+01 | 0.0000 | 4215.0 | 11.50% | 3903.3 | 10.67% | motif file (matrix) | svg |
| 109 | T A C G C T A G A T G C G A T C G T A C A G T C C T A G A G T C A G T C A G T C G T A C A G T C | Sp1(Zf)/Promoter/Homer | 1e-6 | -1.551e+01 | 0.0000 | 728.0 | 1.99% | 600.0 | 1.64% | motif file (matrix) | svg |
| 110 | T A C G T C G A G A C T A C T G C T G A A G T C T C A G G A C T T G A C C T G A | Atf1(bZIP)/K562-ATF1-ChIP-Seq(GSE31477)/Homer | 1e-6 | -1.466e+01 | 0.0000 | 3130.0 | 8.54% | 2868.3 | 7.84% | motif file (matrix) | svg |
| 111 | T G A C C T G A C T A G T C G A C T G A A T G C C G T A A C T G G C A T G T A C G C A T A T C G G C A T A G C T G A T C | PR(NR)/T47D-PR-ChIP-Seq(GSE31130)/Homer | 1e-6 | -1.452e+01 | 0.0000 | 10781.0 | 29.43% | 10340.0 | 28.27% | motif file (matrix) | svg |
| 112 | A T G C G A C T A G C T C T A G C G T A C T A G C G A T C T A G A T C G G A T C | Nkx2.2(Homeobox)/NPC-Nkx2.2-ChIP-Seq(GSE61673)/Homer | 1e-6 | -1.441e+01 | 0.0000 | 9127.0 | 24.91% | 8712.9 | 23.82% | motif file (matrix) | svg |
| 113 | T A G C A G T C T G A C A G T C C T A G A T C G A G T C C A T G T G A C A G T C G T A C A G T C A G T C G C A T C T A G A T C G G C A T A C T G A T C G G A T C | BORIS(Zf)/K562-CTCFL-ChIP-Seq(GSE32465)/Homer | 1e-6 | -1.430e+01 | 0.0000 | 768.0 | 2.10% | 641.0 | 1.75% | motif file (matrix) | svg |
| 114 | T C A G T G A C G T A C T G C A G T A C C T A G G T A C A T G C A G T C G T C A A G T C G A C T | Klf9(Zf)/GBM-Klf9-ChIP-Seq(GSE62211)/Homer | 1e-6 | -1.411e+01 | 0.0000 | 1758.0 | 4.80% | 1565.0 | 4.28% | motif file (matrix) | svg |
| 115 | C G T A C T A G C A T G A G C T A C T G C G A T A T C G C G T A G T C A G T C A | Tbet(T-box)/CD8-Tbet-ChIP-Seq(GSE33802)/Homer | 1e-6 | -1.408e+01 | 0.0000 | 5897.0 | 16.10% | 5554.0 | 15.19% | motif file (matrix) | svg |
| 116 | A T G C C T G A G A C T A C G T A C G T G T A C G A T C C G A T C T A G C A T G C G T A C G T A C T G A G A C T | STAT1(Stat)/HelaS3-STAT1-ChIP-Seq(GSE12782)/Homer | 1e-6 | -1.399e+01 | 0.0000 | 1757.0 | 4.80% | 1565.6 | 4.28% | motif file (matrix) | svg |
| 117 | C A T G G A C T G C A T A C T G A G C T A C T G A C T G C G T A G C A T A G C T A T C G T A C G | Foxh1(Forkhead)/hESC-FOXH1-ChIP-Seq(GSE29422)/Homer | 1e-6 | -1.395e+01 | 0.0000 | 3672.0 | 10.02% | 3397.5 | 9.29% | motif file (matrix) | svg |
| 118 | T A G C T C A G C A T G G C A T A G C T C G A T A T G C C G T A C G T A G T C A | CHR(?)/Hela-CellCycle-Expression/Homer | 1e-6 | -1.392e+01 | 0.0000 | 4342.0 | 11.85% | 4045.7 | 11.06% | motif file (matrix) | svg |
| 119 | C G A T T A C G T G C A G T A C G A T C G A C T A G C T A C G T A T C G G T A C G A T C G T A C G A T C G T C A | PPARE(NR),DR1/3T3L1-Pparg-ChIP-Seq(GSE13511)/Homer | 1e-5 | -1.344e+01 | 0.0000 | 5534.0 | 15.10% | 5209.1 | 14.24% | motif file (matrix) | svg |
| 120 | C T A G T C A G C A G T T C A G A C T G A C T G G A T C C T A G A C T G C T A G T C A G A T G C | KLF14(Zf)/HEK293-KLF14.GFP-ChIP-Seq(GSE58341)/Homer | 1e-5 | -1.293e+01 | 0.0000 | 7826.0 | 21.36% | 7458.6 | 20.39% | motif file (matrix) | svg |
| 121 | T A C G C T G A T C G A C G A T C T A G C T A G T C G A C T G A T C G A T C G A C G T A T C G A G C A T C A T G C G T A T A C G G C A T T G A C C G T A A G C T | NFAT:AP1(RHD,bZIP)/Jurkat-NFATC1-ChIP-Seq(Jolma\_et\_al.)/Homer | 1e-5 | -1.282e+01 | 0.0000 | 1174.0 | 3.20% | 1025.7 | 2.80% | motif file (matrix) | svg |
| 122 | T A G C C T A G T C G A G A C T A C T G C T G A A G T C T C A G G C A T T G A C C T G A A G C T | Atf7(bZIP)/3T3L1-Atf7-ChIP-Seq(GSE56872)/Homer | 1e-5 | -1.277e+01 | 0.0000 | 2218.0 | 6.05% | 2013.8 | 5.51% | motif file (matrix) | svg |
| 123 | C A T G A C T G A G C T A T G C C G T A A T G C G T A C G A C T T A C G C T G A A C T G A C T G G C A T A T G C C T G A | THRb(NR)/HepG2-THRb.Flag-ChIP-Seq(Encode)/Homer | 1e-5 | -1.277e+01 | 0.0000 | 2513.0 | 6.86% | 2295.1 | 6.28% | motif file (matrix) | svg |
| 124 | T G C A T C G A T C G A A C G T C A T G C G T A A G T C T C A G A C G T G T A C C G T A A G C T | CREB5(bZIP)/LNCaP-CREB5.V5-ChIP-Seq(GSE137775)/Homer | 1e-5 | -1.266e+01 | 0.0000 | 2110.0 | 5.76% | 1911.9 | 5.23% | motif file (matrix) | svg |
| 125 | C T G A C T G A T A G C G A T C G C T A G T A C A C G T G A T C T G C A C G T A | Nkx2.5(Homeobox)/HL1-Nkx2.5.biotin-ChIP-Seq(GSE21529)/Homer | 1e-5 | -1.250e+01 | 0.0000 | 10816.0 | 29.52% | 10409.4 | 28.46% | motif file (matrix) | svg |
| 126 | A G C T C T G A C T A G C T A G A C T G T A G C T G C A T C G A C T G A C T A G C A T G A C G T A T G C T C G A | RXR(NR),DR1/3T3L1-RXR-ChIP-Seq(GSE13511)/Homer | 1e-5 | -1.250e+01 | 0.0000 | 6620.0 | 18.07% | 6283.4 | 17.18% | motif file (matrix) | svg |
| 127 | C T G A C T G A C A T G A T C G A G C T A T C G G A C T C A T G C T G A G T C A | Tbr1(T-box)/Cortex-Tbr1-ChIP-Seq(GSE71384)/Homer | 1e-5 | -1.241e+01 | 0.0000 | 7844.0 | 21.41% | 7484.4 | 20.46% | motif file (matrix) | svg |
| 128 | G C T A G A C T G A C T T G C A C G T A A G T C C G T A T A G C G A T C G A C T | Eomes(T-box)/H9-Eomes-ChIP-Seq(GSE26097)/Homer | 1e-5 | -1.230e+01 | 0.0000 | 11992.0 | 32.73% | 11575.1 | 31.65% | motif file (matrix) | svg |
| 129 | T A C G A C T G A G C T G T A C C G T A T C G A C T G A A C T G C A T G A C G T A G T C C G T A | COUP-TFII(NR)/K562-NR2F1-ChIP-Seq(Encode)/Homer | 1e-5 | -1.228e+01 | 0.0000 | 7911.0 | 21.59% | 7552.7 | 20.65% | motif file (matrix) | svg |
| 130 | A C T G G A T C C T G A A T C G A G T C T A G C C T G A C G T A T A C G A G T C C T A G C A G T T C A G T C G A T G A C G A T C | PAX5(Paired,Homeobox)/GM12878-PAX5-ChIP-Seq(GSE32465)/Homer | 1e-5 | -1.164e+01 | 0.0000 | 1484.0 | 4.05% | 1326.0 | 3.63% | motif file (matrix) | svg |
| 131 | A G T C G A C T A C T G G A T C G T A C C G T A T G A C A G T C C G A T A G C T A C G T A C G T C T A G G A C T C T G A | ZNF7(Zf)/HepG2-ZNF7.Flag-ChIP-Seq(Encode)/Homer | 1e-5 | -1.161e+01 | 0.0000 | 2890.0 | 7.89% | 2669.1 | 7.30% | motif file (matrix) | svg |
| 132 | A G T C G C A T C G T A C G T A G T A C A C G T A C T G G A T C G A T C T C G A | BMYB(HTH)/Hela-BMYB-ChIP-Seq(GSE27030)/Homer | 1e-5 | -1.155e+01 | 0.0000 | 7704.0 | 21.03% | 7361.3 | 20.13% | motif file (matrix) | svg |
| 133 | C T G A A T G C C G T A A C G T A G T C A G T C A C G T A C T G A T C G G C A T | SPDEF(ETS)/VCaP-SPDEF-ChIP-Seq(SRA014231)/Homer | 1e-4 | -1.145e+01 | 0.0000 | 6401.0 | 17.47% | 6085.1 | 16.64% | motif file (matrix) | svg |
| 134 | C T A G C A T G A C G T A G T C G C T A A G C T A G T C A G C T T C A G C T G A A C T G C A T G G C A T A T G C C G T A | THRa(NR)/C17.2-THRa-ChIP-Seq(GSE38347)/Homer | 1e-4 | -1.142e+01 | 0.0000 | 1897.0 | 5.18% | 1719.0 | 4.70% | motif file (matrix) | svg |
| 135 | C G T A C G A T G A C T G A C T T G A C C T G A A T G C C T G A T A G C A G T C A C G T T C G A C A T G T A C G G A C T A T C G G A C T A C G T C T G A T C G A C G T A | Brachyury(T-box)/Mesoendoderm-Brachyury-ChIP-exo(GSE54963)/Homer | 1e-4 | -1.142e+01 | 0.0000 | 1442.0 | 3.94% | 1287.7 | 3.52% | motif file (matrix) | svg |
| 136 | A C G T G A C T A T G C G C T A C T G A C T A G A C T G G A C T A G T C C G T A | Nr5a2(NR)/mES-Nr5a2-ChIP-Seq(GSE19019)/Homer | 1e-4 | -1.140e+01 | 0.0000 | 2710.0 | 7.40% | 2498.4 | 6.83% | motif file (matrix) | svg |
| 137 | T C A G A G C T A T G C C G T A A G T C T C A G A C G T A T C G T C G A A G T C G A T C T G A C | TFE3(bHLH)/MEF-TFE3-ChIP-Seq(GSE75757)/Homer | 1e-4 | -1.135e+01 | 0.0000 | 301.0 | 0.82% | 233.9 | 0.64% | motif file (matrix) | svg |
| 138 | A C T G A C G T C A T G A T C G A T C G T G A C A C T G A T C G A T C G T G C A C T G A C G T A | E2F3(E2F)/MEF-E2F3-ChIP-Seq(GSE71376)/Homer | 1e-4 | -1.125e+01 | 0.0000 | 2063.0 | 5.63% | 1879.1 | 5.14% | motif file (matrix) | svg |
| 139 | C G T A T A G C A G T C C T A G C A G T C T A G C T G A G T A C G C A T T C G A C G T A G C A T A G C T C T A G T C G A | PAX3:FKHR-fusion(Paired,Homeobox)/Rh4-PAX3:FKHR-ChIP-Seq(GSE19063)/Homer | 1e-4 | -1.117e+01 | 0.0000 | 1094.0 | 2.99% | 961.5 | 2.63% | motif file (matrix) | svg |
| 140 | T G A C T C G A C T G A C T G A A T G C G A T C C T A G T A C G G A C T G A C T G A T C T C G A C T G A C T G A A T G C G A T C C T A G A T C G G A C T G A C T | Tcfcp2l1(CP2)/mES-Tcfcp2l1-ChIP-Seq(GSE11431)/Homer | 1e-4 | -1.103e+01 | 0.0001 | 581.0 | 1.59% | 486.0 | 1.33% | motif file (matrix) | svg |
| 141 | G A C T T C G A C G A T T A C G C T A G T A C G A C T G A T G C G T A C G T A C | Zac1(Zf)/Neuro2A-Plagl1-ChIP-Seq(GSE75942)/Homer | 1e-4 | -1.090e+01 | 0.0001 | 13031.0 | 35.57% | 12632.6 | 34.54% | motif file (matrix) | svg |
| 142 | T C A G A C G T A G T C T C G A A G T C T C A G G C A T C T A G C T A G A G C T | Usf2(bHLH)/C2C12-Usf2-ChIP-Seq(GSE36030)/Homer | 1e-4 | -1.065e+01 | 0.0001 | 1259.0 | 3.44% | 1120.6 | 3.06% | motif file (matrix) | svg |
| 143 | C A G T T C A G G A T C A C T G A C G T C T A G A C T G A C T G G A C T C T A G | Egr1(Zf)/K562-Egr1-ChIP-Seq(GSE32465)/Homer | 1e-4 | -1.053e+01 | 0.0001 | 2668.0 | 7.28% | 2467.3 | 6.75% | motif file (matrix) | svg |
| 144 | T A G C G A T C G T A C C T G A G C A T C T G A C G T A T G C A C G T A G A T C | Hoxa13(Homeobox)/ChickenMSG-Hoxa13.Flag-ChIP-Seq(GSE86088)/Homer | 1e-4 | -1.046e+01 | 0.0001 | 16465.0 | 44.94% | 16054.7 | 43.90% | motif file (matrix) | svg |
| 145 | C T G A C T A G A C T G G C A T C T A G G C A T A T C G C T G A C G T A G T C A | Tbx21(T-box)/GM12878-TBX21-ChIP-Seq(Encode)/Homer | 1e-4 | -1.039e+01 | 0.0001 | 5472.0 | 14.94% | 5193.0 | 14.20% | motif file (matrix) | svg |
| 146 | T G A C C T A G A C T G T A G C C G A T A C T G A T G C C A T G A T C G A T C G A T C G T A G C C T G A T A G C G C T A A C T G C G T A A G C T C G T A C T G A | GATA:SCL(Zf,bHLH)/Ter119-SCL-ChIP-Seq(GSE18720)/Homer | 1e-4 | -1.007e+01 | 0.0001 | 530.0 | 1.45% | 444.0 | 1.21% | motif file (matrix) | svg |
| 147 | A G T C A G T C C G A T A C G T A C G T A C T G A C G T A G C T A G T C A G T C | Sox4(HMG)/proB-Sox4-ChIP-Seq(GSE50066)/Homer | 1e-4 | -9.842e+00 | 0.0002 | 4721.0 | 12.89% | 4468.8 | 12.22% | motif file (matrix) | svg |
| 148 | C T G A C A G T C T G A A G T C C T A G G A C T A T C G G T A C | HIF-1b(HLH)/T47D-HIF1b-ChIP-Seq(GSE59937)/Homer | 1e-4 | -9.830e+00 | 0.0002 | 3956.0 | 10.80% | 3723.8 | 10.18% | motif file (matrix) | svg |
| 149 | C T G A T A C G G C A T A G C T A G C T A G T C T C G A A C T G C A G T A G C T A G C T G A T C | IRF3(IRF)/BMDM-Irf3-ChIP-Seq(GSE67343)/Homer | 1e-4 | -9.645e+00 | 0.0002 | 2261.0 | 6.17% | 2085.1 | 5.70% | motif file (matrix) | svg |
| 150 | A C G T T A C G G A T C A C T G A C G T C T A G A C T G A C T G G A T C C T A G C A T G C T A G | Egr2(Zf)/Thymocytes-Egr2-ChIP-Seq(GSE34254)/Homer | 1e-4 | -9.620e+00 | 0.0002 | 533.0 | 1.45% | 449.9 | 1.23% | motif file (matrix) | svg |
| 151 | C G T A C T G A A C T G A G C T A G T C G A T C G A T C G C A T C T G A C T A G C T A G T A C G T C G A T G C A G C A T | EBF2(EBF)/BrownAdipose-EBF2-ChIP-Seq(GSE97114)/Homer | 1e-4 | -9.550e+00 | 0.0002 | 5083.0 | 13.87% | 4826.8 | 13.20% | motif file (matrix) | svg |
| 152 | C T A G C T A G A G T C T C A G A C T G A C G T A C G T C T G A | MYB(HTH)/ERMYB-Myb-ChIPSeq(GSE22095)/Homer | 1e-4 | -9.513e+00 | 0.0002 | 8799.0 | 24.02% | 8476.7 | 23.18% | motif file (matrix) | svg |
| 153 | T C A G A T C G G A C T A C T G G A C T C A G T C T A G C G T A G T A C C G T A C T A G A T C G | Tbx20(T-box)/Heart-Tbx20-ChIP-Seq(GSE29636)/Homer | 1e-4 | -9.465e+00 | 0.0002 | 977.0 | 2.67% | 863.8 | 2.36% | motif file (matrix) | svg |
| 154 | G T A C G A T C C A G T A G T C A G T C A G T C T G C A G A T C C T G A A T G C G T C A A C G T | WT1(Zf)/Kidney-WT1-ChIP-Seq(GSE90016)/Homer | 1e-4 | -9.396e+00 | 0.0002 | 2758.0 | 7.53% | 2567.3 | 7.02% | motif file (matrix) | svg |
| 155 | T C G A A C T G C A T G A G C T A G T C C G T A C T G A C T A G A C T G C G A T A T G C C T G A | RAR:RXR(NR),DR0/ES-RAR-ChIP-Seq(GSE56893)/Homer | 1e-3 | -9.122e+00 | 0.0003 | 618.0 | 1.69% | 530.6 | 1.45% | motif file (matrix) | svg |
| 156 | A C T G C G T A A C T G A T G C T G A C G A T C A T C G T G C A A C T G A G T C | ZNF519(Zf)/HEK293-ZNF519.GFP-ChIP-Seq(GSE58341)/Homer | 1e-3 | -9.062e+00 | 0.0003 | 781.0 | 2.13% | 682.3 | 1.87% | motif file (matrix) | svg |
| 157 | T G C A A G C T A C G T C T A G G A T C C T A G G A T C G T C A C T G A A G T C | CEBP(bZIP)/ThioMac-CEBPb-ChIP-Seq(GSE21512)/Homer | 1e-3 | -8.564e+00 | 0.0005 | 3663.0 | 10.00% | 3456.9 | 9.45% | motif file (matrix) | svg |
| 158 | A C T G C T A G G A T C C G T A C T A G A G T C T G C A G A C T C G T A A C G T T C A G A G T C A C G T C T G A A G T C A G T C G A T C C G T A T C A G T A C G | EBNA1(EBV-virus)/Raji-EBNA1-ChIP-Seq(GSE30709)/Homer | 1e-3 | -8.121e+00 | 0.0008 | 64.0 | 0.17% | 40.7 | 0.11% | motif file (matrix) | svg |
| 159 | C T G A C T A G A T C G A G C T A C T G G A C T A G T C C T G A | Tbx5(T-box)/HL1-Tbx5.biotin-ChIP-Seq(GSE21529)/Homer | 1e-3 | -8.078e+00 | 0.0009 | 15939.0 | 43.50% | 15587.3 | 42.62% | motif file (matrix) | svg |
| 160 | A T G C A T C G T A C G A G C T A T C G C T G A A G T C C T A G A G C T A T G C C T G A A T G C | CRE(bZIP)/Promoter/Homer | 1e-3 | -7.613e+00 | 0.0014 | 712.0 | 1.94% | 627.2 | 1.71% | motif file (matrix) | svg |
| 161 | C T A G A C G T G C A T T C G A G T C A G C A T A T C G C G T A C A G T A G C T C T G A T G C A | HNF1b(Homeobox)/PDAC-HNF1B-ChIP-Seq(GSE64557)/Homer | 1e-3 | -7.362e+00 | 0.0017 | 1161.0 | 3.17% | 1054.4 | 2.88% | motif file (matrix) | svg |
| 162 | A C T G A G C T A G T C G T C A A G C T T C A G A T G C G A T C G C A T A T C G T C G A T A G C C G A T C A T G T A G C | Pax8(Paired,Homeobox)/Thyroid-Pax8-ChIP-Seq(GSE26938)/Homer | 1e-3 | -7.301e+00 | 0.0018 | 1399.0 | 3.82% | 1282.4 | 3.51% | motif file (matrix) | svg |
| 163 | C G A T A C G T A C G T A C G T C G T A A G C T C A G T C T A G A T C G A C T G | HOXB13(Homeobox)/ProstateTumor-HOXB13-ChIP-Seq(GSE56288)/Homer | 1e-3 | -7.123e+00 | 0.0022 | 7242.0 | 19.77% | 6991.7 | 19.12% | motif file (matrix) | svg |
| 164 | G A C T A G T C C G A T A C T G C T G A T G A C G T A C C G T A A T C G G C A T C T G A C T A G | Bcl11a(Zf)/HSPC-BCL11A-ChIP-Seq(GSE104676)/Homer | 1e-3 | -7.010e+00 | 0.0024 | 3705.0 | 10.11% | 3521.2 | 9.63% | motif file (matrix) | svg |
| 165 | C G T A C T G A C T A G C G T A C G T A A G T C C G T A C A G T G C A T G T C A C G A T A C T G A C G T G C A T G A T C | PGR(NR)/EndoStromal-PGR-ChIP-Seq(GSE69539)/Homer | 1e-3 | -6.933e+00 | 0.0026 | 1182.0 | 3.23% | 1078.0 | 2.95% | motif file (matrix) | svg |
| 166 | A G C T A C G T A C T G A T G C A G T C C G T A C T G A T A C G | NF1-halfsite(CTF)/LNCaP-NF1-ChIP-Seq(Unpublished)/Homer | 1e-2 | -6.858e+00 | 0.0028 | 9045.0 | 24.69% | 8777.7 | 24.00% | motif file (matrix) | svg |
| 167 | T A C G G A C T A C T G A C T G C T A G A T G C A G T C A G T C A G T C C T G A | ZNF692(Zf)/HEK293-ZNF692.GFP-ChIP-Seq(GSE58341)/Homer | 1e-2 | -6.712e+00 | 0.0032 | 684.0 | 1.87% | 607.8 | 1.66% | motif file (matrix) | svg |
| 168 | C T G A A C G T A C G T A C G T A G T C G A C T C G A T C T G A A C T G C G T A C G T A T C G A | STAT5(Stat)/mCD4+-Stat5-ChIP-Seq(GSE12346)/Homer | 1e-2 | -6.546e+00 | 0.0038 | 2012.0 | 5.49% | 1881.7 | 5.14% | motif file (matrix) | svg |
| 169 | G A C T C T A G C T A G C T A G A C T G T C G A C T G A C T A G C T A G C T A G G T A C G T C A | ZNF467(Zf)/HEK293-ZNF467.GFP-ChIP-Seq(GSE58341)/Homer | 1e-2 | -6.536e+00 | 0.0038 | 4411.0 | 12.04% | 4220.2 | 11.54% | motif file (matrix) | svg |
| 170 | C T G A A G C T A C G T A C G T A G T C G A C T G A C T C T G A C T G A C T A G C G T A C G T A | STAT6(Stat)/CD4-Stat6-ChIP-Seq(GSE22104)/Homer | 1e-2 | -6.420e+00 | 0.0042 | 3397.0 | 9.27% | 3230.3 | 8.83% | motif file (matrix) | svg |
| 171 | A T G C C G T A A C T G C G T A A C G T G C T A T C G A A G C T C G A T C G T A A C G T A G T C C G A T A C T G G A T C | GATA(Zf),IR4/iTreg-Gata3-ChIP-Seq(GSE20898)/Homer | 1e-2 | -6.352e+00 | 0.0045 | 467.0 | 1.27% | 407.0 | 1.11% | motif file (matrix) | svg |
| 172 | G C T A T A G C A G C T A T C G G T C A C G T A G C T A A T G C G A T C C T G A | IRF4(IRF)/GM12878-IRF4-ChIP-Seq(GSE32465)/Homer | 1e-2 | -6.262e+00 | 0.0049 | 2584.0 | 7.05% | 2440.5 | 6.67% | motif file (matrix) | svg |
| 173 | A C T G G A C T A G T C C T G A G A T C T C A G A T G C G A C T A G T C A T G C T A G C A G C T A T C G T G C A | PAX5(Paired,Homeobox),condensed/GM12878-PAX5-ChIP-Seq(GSE32465)/Homer | 1e-2 | -6.211e+00 | 0.0051 | 380.0 | 1.04% | 326.9 | 0.89% | motif file (matrix) | svg |
| 174 | A C G T G A C T T A G C C G T A C T G A C A T G C T A G G A C T G A T C C G T A | Nr5a2(NR)/Pancreas-LRH1-ChIP-Seq(GSE34295)/Homer | 1e-2 | -6.132e+00 | 0.0055 | 3660.0 | 9.99% | 3492.1 | 9.55% | motif file (matrix) | svg |
| 175 | C T A G C T A G A C T G A G C T G A C T A G C T A C G T C T A G G T C A C T G A A C T G T C A G C T G A A C G T C T A G G C T A C T G A A C G T C G T A C T A G A C T G C G T A A C T G A G C T A C G T | ZFP3(Zf)/HEK293-ZFP3.GFP-ChIP-Seq(GSE58341)/Homer | 1e-2 | -6.102e+00 | 0.0056 | 29.0 | 0.08% | 16.9 | 0.05% | motif file (matrix) | svg |
| 176 | C T A G A C T G C T A G T C A G T C A G T A C G C T A G A C T G | Maz(Zf)/HepG2-Maz-ChIP-Seq(GSE31477)/Homer | 1e-2 | -6.011e+00 | 0.0061 | 6334.0 | 17.29% | 6121.9 | 16.74% | motif file (matrix) | svg |
| 177 | A T G C A G T C A G C T A G C T A C G T A T C G C G T A C G A T T A G C G A C T | LEF1(HMG)/H1-LEF1-ChIP-Seq(GSE64758)/Homer | 1e-2 | -5.532e+00 | 0.0098 | 4065.0 | 11.10% | 3900.2 | 10.66% | motif file (matrix) | svg |
| 178 | C T G A T C A G C T G A C T A G C A T G A C G T A T G C C G T A A T G C G C A T T C A G C T G A A C T G A C G T C A G T A G T C C G T A C A G T C T A G C A T G | VDR(NR),DR3/GM10855-VDR+vitD-ChIP-Seq(GSE22484)/Homer | 1e-2 | -5.491e+00 | 0.0102 | 1045.0 | 2.85% | 961.9 | 2.63% | motif file (matrix) | svg |
| 179 | C A T G A C G T A G T C G A T C G A T C G A T C G C T A C T A G C T A G C T A G T C A G T C G A | EBF1(EBF)/Near-E2A-ChIP-Seq(GSE21512)/Homer | 1e-2 | -5.410e+00 | 0.0110 | 5513.0 | 15.05% | 5326.7 | 14.56% | motif file (matrix) | svg |
| 180 | A T C G G A C T C G A T C G T A C G T A C A G T G A T C G A T C G A T C A G C T | Otx2(Homeobox)/EpiLC-Otx2-ChIP-Seq(GSE56098)/Homer | 1e-2 | -5.340e+00 | 0.0117 | 5039.0 | 13.75% | 4862.0 | 13.29% | motif file (matrix) | svg |
| 181 | T G A C C T A G T C A G G T C A C G T A T C A G C G A T T C A G T C G A T G C A C T G A T A G C | PU.1-IRF(ETS:IRF)/Bcell-PU.1-ChIP-Seq(GSE21512)/Homer | 1e-2 | -5.252e+00 | 0.0127 | 8258.0 | 22.54% | 8040.5 | 21.98% | motif file (matrix) | svg |
| 182 | A T G C A G C T T C A G T G A C T C A G A T G C T G C A A C G T A T C G G A T C A C T G A G T C | NRF1(NRF)/MCF7-NRF1-ChIP-Seq(Unpublished)/Homer | 1e-2 | -5.155e+00 | 0.0140 | 177.0 | 0.48% | 145.1 | 0.40% | motif file (matrix) | svg |
| 183 | C T G A C T G A C T A G T C G A C G T A A T G C C G T A A C T G C G T A A C G T C T G A C G A T A G C T C G T A A C G T A G T C C G A T T A C G G T C A G C A T | GATA(Zf),IR3/iTreg-Gata3-ChIP-Seq(GSE20898)/Homer | 1e-2 | -5.146e+00 | 0.0140 | 819.0 | 2.24% | 748.5 | 2.05% | motif file (matrix) | svg |
| 184 | C G T A C T A G G A C T G T C A G T C A C G T A A G T C C G T A T C G A T C G A T C G A C G T A C T G A C T A G G C T A C G T A T A G C C G T A C G A T C G T A | FOXA1:AR(Forkhead,NR)/LNCAP-AR-ChIP-Seq(GSE27824)/Homer | 1e-2 | -5.098e+00 | 0.0146 | 541.0 | 1.48% | 484.1 | 1.32% | motif file (matrix) | svg |
| 185 | C A T G A G T C C T G A T G A C A T C G G A C T G T C A A G T C T A G C G A T C | HIF2a(bHLH)/785\_O-HIF2a-ChIP-Seq(GSE34871)/Homer | 1e-2 | -5.072e+00 | 0.0149 | 1302.0 | 3.55% | 1213.8 | 3.32% | motif file (matrix) | svg |
| 186 | C T G A T C A G G C T A A G C T A G T C G A C T C T G A C T A G T G C A C T G A A G T C G T A C G A T C A C T G T C G A | ZBTB12(Zf)/HEK293-ZBTB12.GFP-ChIP-Seq(GSE58341)/Homer | 1e-2 | -5.034e+00 | 0.0154 | 1971.0 | 5.38% | 1862.9 | 5.09% | motif file (matrix) | svg |
| 187 | C T A G T C G A C G A T C T A G G C A T C A G T C T A G G A T C C G T A G T C A | CEBP:AP1(bZIP)/ThioMac-CEBPb-ChIP-Seq(GSE21512)/Homer | 1e-2 | -4.967e+00 | 0.0164 | 4152.0 | 11.33% | 3997.1 | 10.93% | motif file (matrix) | svg |
| 188 | A T G C A T G C A T C G T A C G A G C T A G T C G C T A A G T C T C A G G A C T A C T G T C G A | E-box(bHLH)/Promoter/Homer | 1e-2 | -4.944e+00 | 0.0167 | 221.0 | 0.60% | 186.2 | 0.51% | motif file (matrix) | svg |
| 189 | T C G A C A T G C A T G T A C G G T A C T C G A A G T C C A G T C T G A C G T A A G T C G A C T | ZNF264(Zf)/HEK293-ZNF264.GFP-ChIP-Seq(GSE58341)/Homer | 1e-2 | -4.806e+00 | 0.0191 | 2778.0 | 7.58% | 2654.0 | 7.26% | motif file (matrix) | svg |
| 190 | T C G A A C G T A C G T C T G A G A T C T C A G G A C T G T C A C G T A A G C T G T C A C T A G A G C T A C G T T C G A | NFIL3(bZIP)/HepG2-NFIL3-ChIP-Seq(Encode)/Homer | 1e-2 | -4.721e+00 | 0.0206 | 3769.0 | 10.29% | 3626.5 | 9.92% | motif file (matrix) | svg |
